# Post-COVID-19 pulmonary complications among recovered COVID-19 patients: a cross-sectional study from Addis Ababa, Ethiopia

**DOI:** 10.1186/s12890-023-02731-x

**Published:** 2023-10-27

**Authors:** Abebaw Bekele Seyoum, Sisay Shine Tegegnework, Mahider Molla Mengistu, Tenbite Daniel Mekonnen, Abdurahman Mohammedamin Asabel, Alazar Gizate Dagnaw, Abenet Girma Deribe, Tadios Niguss Derese, Tsegaye Gebreyes Hundie, Bisrat Kassa Getahun, Dawit Kebede Huluka

**Affiliations:** 1 Eka Kotebe General Hospital, Addis Ababa, Ethiopia; 2 Debre Berhan University, Debre Birhan, Ethiopia; 3 Addis Ababa University, Addis Ababa, Ethiopia

**Keywords:** COVID-19, Post COVID-19 pulmonary Complications, Eka Kotebe General Hospital, Addis Ababa, Ethiopia

## Abstract

**Background:**

The COVID-19 pandemic has been linked to chronic pulmonary complications all over the world. Respiratory complications such as chronic cough, dyspnea, increased respiratory rate, and oxygen support demand are prevalent in recovered COVID-19 patients. These problems are long-term and have a negative impact on one’s quality of life. Patients must be evaluated for potential complications, and risk factors must be found. Some reports around the world explain the factors that contribute to the development of these complications. However, to the best of our understanding, no reports of post-COVID-19 complications have been reported from Ethiopia.

**Methods:**

Facility based cross-sectional study was done among 405 participants selected by simple random sampling technique. Structured questionnaire which includes participants’ demographic, clinical and 3rd month visit characteristics was collected by Open Data Kit and exported to SPSS version 25.0 for analysis. Percentage with frequency and median with Interquartile range was used in descriptive statistics. The association between variables was analyzed with bivariate and multi variable logistic regression. A statistical significance was declared at p-value < 0.05, with 95% confidence interval.

**Results:**

The median (Interquartile range) age of participants was 57.0 (43.0, 65.0) years, 63.2% were males. The prevalence of post-COVID-19 pulmonary complication in recovered COVID-19 patients was 14.1% (95% CI: 10.8%, 17.8%). After adjusting for possible confounders on multivariate analysis, older age [AOR = 0.227, 95% CI (0.08–0.66)] and consolidation [AOR = 0.497, 95% CI (0.258–0.957)] were shown to have significant association with post COVID-19 pulmonary complications.

**Conclusion:**

The prevalence of post COVID-19 pulmonary complication was observed to be lower than other reports globally. Older age and the presence of consolidation on lung imaging were associated with those complications. Clinicians are recommended to consider assessing the lasting effects of the pandemic, beyond immediate care, and should also investigate the COVID-19 history in patients presenting with respiratory issues.

## Background

The COVID-19 pandemic is observed to have long-lasting impacts on the respiratory system, which is primarily affected in the acute phase of infection. In recovering patients, it is common to observe a cough, shortness of breath, decreased respiratory functions, a need for oxygen support, and radiologic evidence of chronic lung inflammation [1–4].

The care and follow-up of infected patients should continue after the acute phase, as an important proportion of patients continue to have persistent symptoms and complications, as can be learned from the previous coronavirus-originating epidemics [5–7]. Given that this pandemic impacted a considerably higher number of patients, the post-acute phase of the pandemic presents a significant challenge, as is demonstrated in a number of reports from around the world [8–11].

Post COVID-19 pulmonary complications have been reported around the globe. Multiple reports from US, Italy and other countries suggest that the prevalence of these symptoms is high, some reporting as high as 87.4% [12–14]. Fatigue, shortness of breath, decreased activity and other psychological effects are seen in these patients. Additional diagnosis and even death were reported during follow-ups of recovered patients in some reports, which were attributed to the less anticipated post-acute infection complications of the virus [4, 14]. The Egypt study done on female survivors, with a result of 77.4% prevalence of post-COVID-19 symptoms is the only one from our continent [15]. Female gender, Intensive Care Unit admission, presence of comorbidities and treatments given during the acute phase are some of the factors outlined in the reports [9, 10, 16–18]. To the best of our knowledge, there are no reports done in Ethiopia so far.It will be essential to describe the prevalence and factors linked with these complications in order to reduce the morbidity and mortality caused by the post-acute phase of the pandemic. There is a research gap in this topic from low income countries like Ethiopia and this study aims to provide some evidence.

## Methods

### Study design and setting

Facility based cross sectional study design was conducted to assess the prevalence and associated factors of post COVID-19 pulmonary complications among recovered COVID-19 patients in Eka Kotebe General Hospital, which is the largest treatment centre in the country. The hospital had post-COVID follow up clinic which is run by general practitioners, consultant internists and a pulmonologist. The data was taken from the medical records of patients who, between January 2021 and January 2022, had their third month follow-up appointment after being admitted with confirmed COVID-19 and were discharged as recovered.

### Sampling procedure and eligibility criteria

The sample size was calculated by using single population formula with the assumptions of: 95%CI, 5% margin of error, with 40.2% expected proportion [10]. 10% was added accounting for chart loss, which gives a total of 405. All patients whose age was greater than 18 years were included in the study. In the months of January 2021 and January 2022, 1573 people went to the post-COVID-19 Out Patient Department at Eka Kotebe General Hospital. 763 of these individuals underwent visits during the third month of follow-up. Using the Microsoft Excel application, a simple random sampling process was carried out.

### Data collection and quality assurance

The data was collected by using pretested structured checklist which is adopted from reviewing different related literatures [10, 14, 17, 19]. The checklist had 4 major sections. These were socio-demographic, acute phase and 3rd month clinical condition with comorbidities, treatment given and imaging & lab profiles. The data included the dates of onset of symptoms, admission status and date of discharge. Patients’ admission clinical status and their 3rd month follow-up data was collected from their chart. Trained general practitioners collected the data by an electronic data collection tool ODK (Kobo tool box). Questionnaires were pre-tested.

### Operational definitions

Presence of 2 or more of persistent cough, persistent shortness of breath, increased respiratory rate (> 22 breaths per minute) and requirement of oxygen support (SpO2 < 90% on room air) was used to define post-COVID-19 pulmonary complication during 3rd month visit [8, 13, 19]. The definition and classification of COVID-19 and discharge criteria was primarily based on the national COVID-19 clinical management guideline [20]. Severe COVID-19; defined by any of oxygen saturation < 90%, increased respiratory rate (respiratory rate > 30) and signs of respiratory distress. Critical COVID-19 was defined by the criteria for acute respiratory distress syndrome (ARDS), sepsis, septic shock that will require the provision of life sustaining therapies. Time based recovery: Declaring recovery considering days after the day of lab confirmed diagnosis of COVID-19. Symptom based recovery: Declaring recovery considering days after being asymptomatic (fever and/or cough) in a patient with lab confirmed diagnosis of COVID-19 with moderate, severe or critical illness. If cough is the only symptom judgment should be left for clinician decision. Test based recovery: Declaring recovery considering two negative lab tests done 24 h apart in a patient with lab confirmed diagnosis of COVID-19.

### Statistical analysis

Data was collected using ODK software and, transported to and analyzed using SPSS software version 25.0. Categorical variables were presented as frequency and percentages (%), and continuous variables were described using the median and interquartile range, after checking for skewness. The association of independent variables with the outcome variable was investigated using binary logistic regression analysis. The variables that showed an association with the outcome variable at the bi-variable analysis with p value < 0.25 were entered into the final multivariable analysis to control for potential confounders. Adjusted odds ratio (AOR) along with 95% confidence interval was estimated to assess the strength of association and a P value < 0.05 was considered to declare the statistical significance in the multivariable analysis. Assumptions like model fitness was checked to be satisfied using Hosmer and lemeshow p-value (which should not be significant, i.e. p – value > 0.05).

## Results

### Socio-demographic characteristics of the participants

A total of 405 study participants were involved, 256 (63.2%) were males. The median (IQR) age of the participants was 57.0 (43.0, 65.0) years (Table 1).

**Table 1:** Socio-demographic and presenting symptoms among recovered COVID-19 patients in Eka Kotebe General Hospital; 2022 (n = 405)

Variable	Category	Frequency	Percent (%)
**Age in years**	20–40	88	21.7
	41–60	168	41.5
	> 60	149	36.8
**Sex**	Male	256	63.2
	Female	149	36.8
**Symptoms During Presentation**	Yes	398	98.3
	No	7	1.7
**Cough**	Yes	352	86.9
	No	46	11.4
**Sneezing**	Yes	3	0.7
	No	395	97.5
**Runny Nose**	Yes	7	1.7
	No	391	96.5
**Fever**	Yes	215	53.1
	No	183	45.2
**Headache**	Yes	140	34.6
	No	258	63.7
**Sore throat**	Yes	19	4.7
	No	379	93.6
**Loss of appetite**	Yes	222	54.8
	No	176	43.5
**Shortness of breath**	Yes	187	46.2
	No	211	52.1
**Generalized Fatigue**	Yes	281	69.4
	No	117	28.9
**Diarrhoea or Vomiting**	Yes No	66 332	16.3 82.0
**Loss of taste/Smell**	Yes	79	19.5
	No	319	78.8

### Admission status of participants

Majority of the participants were symptomatic during admission. Cough (86.9%), easy fatigability (69.4%), loss of appetite (54.8%), and fever (53.1%) were the most common symptoms presentation at admission. Shortness of breath and headache were also recorded in 46.2% and 34.6% of participants respectively (Table 1).

### Comorbidities and imaging findings

56.5% of participants had at least one comorbidity. Diabetes Mellitus and hypertension were the most common comorbid illnesses during the time of admission, being present in 35.3% and 30.4% of participants respectively.

Chest imaging (X-ray or CT- Scan) was done for 322 (79.5%) participants. The most common findings were ground glass opacities (GGO) and consolidation. Consolidation was found in 159 (39.3%) of participants, while GGO was present in 274 (67.7%). Infiltration and effusion were also found in 69 (17.0%) and 7 (1.7%) participants respectively (Table 2).

**Table 2:** Comorbidities and imaging findings of participants among recovered COVID-19 patients in Eka Kotebe General Hospital; 2022 (n = 405)

Variable	Category	Frequency	Percent
**Cardiac Illness**	Yes	34	8.4
	No	371	91.6
**CKD**	Yes	13	3.2
	No	392	96.8
**Hypertension**	Yes No	123	30.4
		282	69.6
**DM**	Yes	143	35.3
	No	262	64.7
**Asthma**	Yes	21	5.2
	No	384	94.8
**Smoking**	Yes No	2 403	0.5 99.5
**Malignancy**	Yes	1	0.2
	No	404	99.8
**HIV**	Yes No	14 391	3.5 96.5
**Consolidation**	Yes	159	39.3
	No	246	60.7
**GGO**	Yes	274	67.7
	No	131	32.3
**Infiltration**	Yes	69	17.0
	No	336	83.0
**Effusion**	Yes	7	1.7
	No	398	98.3
**Cardiomegaly**	Yes	1	0.2
	No	404	99.8

### Laboratory parameters

The median (IQR) hemoglobin level was 14.6 g/dl (13.3, 15.8) and the WBC count was 7530 (5340, 10,840) cells per microliter. More than half (51.4%) of participants had a low lymphocyte count (ALC) of less than 1000 per microliter. The median (IQR) lymphocyte count was 973 (656, 1437) cells per microliter. The median (IQR) creatinine and BUN level were 0.91 (0.74, 1.12) mg/dl and 14.0 (10.0, 21.0) mg/dl respectively (Table 3).

**Table 3:** Laboratory parameter during admission and 3rd month visit of participants among recovered COVID-19 patients in Eka Kotebe General Hospital; 2022 (n = 405)

Laboratory Parameters	Admission	3rd month visit
Hemoglobin in g/dl: Median (IQR)	14.6 (13.3,15.8)	15.0 (14.0, 16.2)
WBC Count in cells/microliter: Median (IQR)	7530 (5340, 10,840)	6530 (5010, 8290)
Neutrophil Percentage: Median (IQR)	81.4 (71.9, 87.6)	57.7 (48.7, 65.5)
Lymphocyte percentage: Median (IQR)	12.6 (8.0, 19.8)	28.9 (21.2, 36.8)
ALC per microliter: Median (IQR)	973 (656, 1437)	1776 (1294, 2343)
ALC < 1000 per microliter: Frequency (%)	208 (51.4)	32 (7.9)
ALC $\ge$1000 per microliter: Frequency (%)	197 (48.6)	373 (92.1)
Platelet per microliter: Median (IQR)	211,000 (159,000, 282,000)	212,000 (167,500, 267,500)
Creatinine in mg/dl: Median (IQR)	0.91 (0.74, 1.12)	0.80 (0.67, 1.00)
BUN in mg/dl: Median (IQR)	14.0 (10.0, 21.0)	10.0 (7.0, 14.0)
AST in mg/dl: Median (IQR)	33.0 (21.5, 49.0)	18.0 (14.0, 23.0)
ALT in mg/dl: Median (IQR)	29.0 (12.0, 48.0)	26.0 (18.0, 36.0)

### Clinical course of participants during admission

Among the 405 participants, 327 (80.7%) had severe COVID-19 diagnosis during their hospital stay. Twenty eight (6.9%) had critical COVID-19, 29 (7.2%) had moderate COVID-19 and 21 (5.2%) had mild COVID-19. Majority (87.7%) were admitted to the medical ward while 8.9% of participants admitted to ICU/HDU. The remaining 14 (3.5%) participants were admitted to other wards like the labor ward or psychiatry ward.

Participants received different levels of oxygen support during their stay. Forty seven (11.6%) participants were maintaining their oxygen saturation with room air, without any support, 70.4% of them were supported with 1-5 L per minute of oxygen, 47 (11.6%) participants were given 6-15 L per minute while 5.2% (n = 21) were put on non-invasive ventilation (NIV) support. Only 5 (1.2%) of participants were put on a mechanical ventilator (MV). Steroid was given to 92.1% (n = 373) of participants and 86 (21.2%) participants received antiviral treatment for COVID-19.

The median (IQR) length of stay was 11 [8, 15] days, with a range of 45 days (3–48). When discharged, symptom-based discharge criteria was used for 214 (52.8%) participants. 176 (43.5%) and 15 (3.7%) participants were discharged according to time-based and test-based criteria respectively (Table 4).

**Table 4:** Clinical Course of participants among recovered COVID-19 patients in Eka Kotebe General Hospital; 2022 (n = 405)

Variables	Category	Frequency	Percent (%)
**COVID-19 Severity**	Mild	21	5.2
	Moderate	29	7.2
	Severe	327	80.7
	Critical	28	6.9
**Admission place**	ICU/HDU	36	8.9
	Medical ward	355	87.7
	Other ward	14	3.5
**Steroid given**	Yes	373	92.1
	No	32	7.9
**Length of stay in days**	$\le$14	294	72.6
	15–28	93	23.0
	$>$29	18	4.4
**Maximum Oxygen support**	Room air	47	11.6
	1–5 L	285	70.4
	6–15 L	47	11.6
	NIV	21	5.2
	MV	5	1.2
**Antiviral given**	Yes	86	21.2
	No	319	78.8
**Discharge Criteria**	Time based	176	43.5
	Symptom based	214	52.8
	Test based	15	3.7

### Prevalence of post-covid-19 pulmonary complication

The median (IQR) number of days elapsed from the date of discharge to the visit day was 88 (78, 93) days. During this visit, 72 (17.8%) participants had respiratory complaints. 57(14.1%) had shortness of breath and 31(7.7%) had a cough. Among these symptomatic patients, 16 (4%) participants complained of both cough and shortness of breath, while 56 (13.8%) of them had only one respiratory complaint.

The respiratory rate of participants during the 3rd month visit ranged from 10 to 38 breaths per minute, with a mean number of 22.29 (SD = 2.87), and participants with a respiratory rate of more than 22 breaths per minute were 42.2% (n = 171) of all participants. Oxygen saturation measured at room air during the visit also ranged from 72 − 99%, with a mean measure of 95.54% (SD = 2.74). 23 (5.7%) participants had an oxygen saturation level of less than 90% (Table 5).

**Table 5:** 3rd month visit status of participants for Prevalence and associated factors of Post-COVID-19 Pulmonary Complications among recovered COVID-19 patients in Eka Kotebe General Hospital; 2022 (n = 405)

Variables	Category	Frequency	Percent (%)
Symptom at 3rd month	Yes	72	17.8
	No	333	82.2
Cough	Yes	31	7.7
	No	374	92.3
Shortness of breath	Yes	57	14.1
	No	348	85.9
RR: Median (IQR)	RR $>$22	171	42.2
	RR $\le$22	234	57.8
SpO_2_	SpO_2_ < 90	23	5.7
	SpO_2_$\ge$90	382	94.3
Number of pulmonary complications	1	135	33.3
	2	31	7.7
	3	19	4.7
	4	7	1.7
Consolidation on imaging	Yes	3	0.7
	No	402	99.3
GGO	Yes	19	4.7
	No	386	95.3
Infiltration	Yes	3	0.7
	No	402	99.3
Cardiac illness	Yes	10	2.5
	No	395	97.5
CKD	Yes	8	2.0
	No	397	98.0
Hypertension	Yes	38	9.4
	No	367	90.6
DM	Yes	47	11.6
	No	358	88.4
Asthma	Yes	1	0.2
	No	404	99.8
Smoking	Yes	1	0.2
	No	404	99.8
Stroke	Yes	0	0
	No	405	100
Malignancy	Yes	1	0.2
	No	404	99.8
HIV	Yes	1	0.2
	No	404	99.8

When we take these four pulmonary complications (presence of cough, presence of shortness of breath, increased respiratory rate, and lower oxygen saturation level), 135 (33.3%) of participants had one or more of these complications. Two of the complications were present in 7.7% (n = 31) of participants, while 19 (4.7%) had three complications. 1.7% (n = 7) of participants had all the above four complications. The proportion of participants with 2 or more pulmonary complications which is used to define the prevalence of post COVID-19 pulmonary complications is 14.1% (n = 57) with 95% CI: 10.8%, 17.8%.

### 3rd month visit status of participants

New onset comorbid illnesses, which were not diagnosed during admission were also reported during this period, the most common being DM and hypertension. They were reported in 11.6% (n = 47) and 9.4% (n = 38) of participants respectively. Cardiac disease was found in 10 (2.5%) and CKD was diagnosed in 8(2.0%). GGO was found in 19 (4.7%) and consolidation was seen in 3(0.7%) of participants (Table 5).

The median (IQR) WBC count during 3rd month visit was 6530 (5010, 8290) cells per microliter. Participants with lymphocyte count less than 1000 cells per microliter were 7.9% (n = 32). The median (IQR) lymphocyte count was 1776 (1294, 2343) cells per microliter (Table 3).

### Associated factors of post-COVID-19 pulmonary Complications

In bivariate analysis, p value of < 0.25 was used to determine association between the dependent and independent variables. From demographic characteristics, older age showed significant association with post-COVID-19 pulmonary complications.

From factors in admission status of participants, ALC < 1000 cells per microliter, consolidation, GGO, and infiltration in chest imaging showed significant association with developing pulmonary complications.

When we take variables related with clinical course of participants during admission, COVID severity, admission place, maximum oxygen support level and steroid use were the variables that showed significant association in bivariate analysis.

After adjusting for possible confounders on multivariate analysis, age and consolidation have significant association with the dependent variable at 95% CI (p < 0.05).

Participants whose age is less than 40 years are 77.3% less likely to develop post-COVID-19 pulmonary complications than those aged greater than 60 years [AOR = 0.23, 95% CI (0.08–0.66)]. There is also a 59.9% reduced odds of developing complications in participants aged from 41 to 60 years [AOR = 0.401, 95% CI (0.21–0.79)]. Participants whose chest imaging did not show consolidation were 50.3% less likely to develop pulmonary complications after discharge than their counterparts [AOR = 0.497, 95% CI (0.258–0.957) (Table 6).

**Table 6:** Bivariate and multivariate analysis associated factors of Post-COVID-19 Pulmonary Complications among recovered COVID-19 patients in Eka Kotebe General Hospital; 2022 (n = 405)

Variable	Category	Pulmonary complication		COR (95%CI)	AOR (95%CI)	P value
		Yes	No			
Age in years	20–40	5	83	0.220 (0.082–0.589)	0.227 (0.08–0.66)	0.006
	41–60	20	148	0.49 (0.27–0.91)	0.401 (0.21–0.79)	0.008
	> 60	32	117	1	1	
ALC per microliter	< 1000	34	174	1		
	$\ge$1000	23	174	0.676 (0.383–1.195)	0.870 (0.46–1.63)	0.660
Consolidation	Yes	34	125	1		
	No	23	223	0.379 (0.214–0.672)	0.497 (0.258–0.957)	0.036
GGO	Yes	45	229	1		
	No	12	119	0.513 (0.261–1.007)	0.744 (0.352–1.570)	0.437
Infiltration	Yes	14	55	1		
	No	43	293	0.577 (0.296–1.125)	0.582 (0.276–1.230)	0.156
COVID severity	Mild-Moderate	2	48	0.056 (0.011–0.275)	0.310 (0.006–15.340)	0.556
	Severe	43	284	0.202 (0.089–0.456)	0.493 (0.032–7.683)	0.614
	Critical	12	16	1		
Admission Place	Ward	44	325	0.240 (0.113–0.507)	0.769(0.151–3.903)	0.751
	ICU/HDU	13	23	1		
Maximum Oxygen Support	Room air	2	45	0.061 (0.012–0.305)	1.313 (0.028–61.650)	0.890
	1–15 L/min	44	288	0.208 (0.090–0.483)	1.132 (0.086–14.954)	0.925
	NIV/MV	11	15	1		
Steroid use	Yes	56	317	1		
	No	1	31	0.183 (0.024–1.365)	0.521 (0.030–9.029)	0.654

## Discussion

In this study, the prevalence of post-COVID-19 pulmonary complications was assessed on a median of 88 days after hospital discharge, which is close to the 3 month (90 days) cut point used to define long- COVID-19 in most literatures. These literatures showed a higher prevalence of post COVID-19 complications, up to 39% prevalence report [8, 9, 19, 21]. In our study, cough was present in 7.7% of participants. This was comparable to the Egypt study, which reported 8.7% cough prevalence in recovered patients at 2 month visit post recovery [15]. According to the multicenter study done in Spain, at 11.2 months 2.5% cough prevalence was reported. This low prevalence might be due to the much longer visit day after discharge, compared to this study [18]. A higher prevalence of cough (43%) was also reported in the US study that evaluated patients at 16 days after testing date [22].

Shortness of breath on the day of visit was observed in 14.1% participants, which was close to the Egypt study that reported 17.4% [15]. It is much lower than the study in US, which reported a 29% prevalence, while it is higher than the Spain study that reported a 6.5% prevalence [18, 22].

Compared to majority of reports, this study has a lower prevalence rate of pulmonary complications. A meta-analysis on long COVID by A.V.Raveendran et al. reported around 35% prevalence of residual symptoms at 3 months follow up [8]. A 32% prevalence of 2 complications was also reported in one study [13]. In another study a 40.2% prevalence was reported [10].

New onset comorbidities, which were not recorded during admission, were observed in participants during the visit, DM and hypertension being the commonest ones with 11.6% and 9.4% prevalence. Cardiac illnesses were observed in 2.5% participants. This is comparable to other studies which reported new onset comorbid illnesses after COVID infection. A systematic review and meta-analysis on new onset diabetes reported 19.7% new-onset DM and 25.23% hyperglycemia prevalence [23]. In another Turkey study, 12% new onset hypertension was reported during post-COVID-19 period [24].

Regarding the factors associated with development of pulmonary complications, age greater than 60 years was observed to be at more risk than those lower than that age. There was a 77.3% less chance of pulmonary complications in participants whose age is less than 40 years and 59.8% reduced odd of developing complications in participants aged from 41 to 60 years. This finding was similar to some studies globally, which reported older age as a risk factor for pulmonary complications [9, 10, 22, 25]. In one study with more than 4000 COVID-19 survivors, symptoms lasting more than 28 days were significantly associated with age, rising from 9.9% in the individuals aged 18–49 years to 21.9% in those aged ≥ 70 years [25]. Others also found that, persistent symptoms were common in older age group [9, 10]. In the US study, participants whose age is $\ge 50$ years were 2.29 times more associated with post COVID-19 symptoms [22]. A cross sectional study in Japan also reported an increased risk of long term COVID-19 complications in older age population [26]. This can be due to decreased pulmonary function associated with age increment.

Among the admission status of participants, presence of consolidation was the one factor that showed significant association with post-COVID-19 pulmonary complication, participants with no consolidation in their chest imaging being protected by 50.3%. Extensive chest imaging finding were observed to be associated with pulmonary complications in other studies too [27]. Patients with finding of consolidation and subsequent effect on the lung parenchyma were associated with lasting effects of COVID-19 in this study. Other factors, like number of symptoms, comorbidities and laboratory parameters didn’t show a significant association in this study.

## Conclusion

The prevalence of post-COVID-19 pulmonary complication in recovered COVID-19 individuals is lower than reported elsewhere. Age > 60 years and consolidation in chest imaging have a significant association with pulmonary complications. Clinicians must evaluate the long-term impact of the pandemic, in addition to acute management, and look for a history of COVID-19 infection in patients with pulmonary complaints.

## Data Availability

The datasets used and/or analysed during the current study are available from the corresponding author on reasonable request.

## Abbreviations

ALC: Absolute Lymphocyte Count
BUN: Blood Urea Nitrogen
CI: Confidence Interval
CKD: Chronic Kidney Disease
COVID-19: Coronavirus Disease 2019
DM: Diabetes Mellitus
GGO: Ground Glass Opacities
HDU: High Dependency Unit
ICU: Intensive Care Unit
IRB: Institutional Review Board
MERS-CoV: Middle East Respiratory Syndrome; Coronavirus
MV: Mechanical Ventilator
NIV: Non-Invasive Ventilation
OR: Odds Ratio
SARS-CoV: Severe Acute Respiratory Syndrome; Coronavirus
WBC: White Blood Cell
